# Is artificial intelligence really a new topic in medical education?

**DOI:** 10.3325/cmj.2021.62.200

**Published:** 2021-04

**Authors:** Zdenko Sonicki, Josipa Kern

To the Editor: In the October 2020 issue of the *Croatian Medical Journal*, we read with interest a short communication entitled The Importance of Introducing Artificial Intelligence to the Medical Curriculum – Assessing Practitioners` Perspectives (1). This high-quality short communication on an interesting and important topic presented the results of a national-level survey on Croatian radiologists and radiology residents. The study results support the idea that medical schools should keep pace with the technological progress in medicine by including into their curricula education on artificial intelligence (AI). This general conclusion is even more applicable to Croatian medical schools as it was derived from a survey conducted in Croatia. Although it is out of the research scope of the mentioned paper, to avoid misunderstandings, we must add some missing details on the AI education at Croatian medical schools, specifically the University of Zagreb School of Medicine.

Artificial intelligence is a wide-ranging branch of computer science concerned with the ability of a digital computer or computer-controlled robot to perform tasks that typically require human intelligence. Research in AI has focused chiefly on the following components of intelligence: learning, reasoning, problem solving, perception, and using language.

Schools of Medicine in Croatia (Universities of Zagreb, Rijeka, Osijek, and Split) included AI approach into their medical curriculum through the medical decision-making topic (course: Medical Informatics) in 2009 (2). The School of Medicine in Zagreb teaches this course in the fifth year of study. In addition, knowledge-discovery methodologies, such as machine learning, neural networks, etc, are included in the methodological part of the PhD program (3), as well as into a specific course entitled Knowledge Discovery in Medical Domains, both offered as elective courses since 2005. Students show interest in both courses, as evident in the number of attendees, with 85 students attending the specific knowledge discovery course and 248 attending the methodological course. Furthermore, about twenty doctoral, master's, and student theses with AI-related topics have been defended at the School of Medicine in Zagreb.

Finally, we strongly support the opinion of the authors of the paper, but we also believe that the conclusion about the need to introduce AI into the medical curriculum could be biased due to the inclusion of radiologists and radiology residents only. This means that that the conclusion should be modified to imply the need for the inclusion of AI in the specialist study of radiology.
